# Targeted Stimulation of Micropores by CS_2_ Extraction on Molecular of Coal

**DOI:** 10.3390/molecules29132993

**Published:** 2024-06-23

**Authors:** Zhen Zhang, Gaofeng Liu, Xiaoming Wang, Jia Lin, George Barakos, Ping Chang

**Affiliations:** 1 School of Resources & Environment, Henan Polytechnic University, Jiaozuo 454003, China; 15713944947@163.com; 2 WA School of Mines: Minerals, Energy and Chemical Engineering, Curtin University, Kalgoorlie, WA 6430, Australia; jia.lin@curtin.edu.au (J.L.); george.barakos@curtin.edu.au (G.B.); 3 Collaborative Innovation Center of Coal Work Safety and Clean High Efficiency Utilization, Henan Polytechnic University, Jiaozuo 454003, China; 4 Key Laboratory of Tectonics and Petroleum Resources, China University of Geosciences, Wuhan 430074, China; sunwxm@cug.edu.cn

**Keywords:** carbon disulfide extraction, targeted stimulation, molecular structure simulation, microcrystalline structure evolution, micropore evolution

## Abstract

The targeted stimulation of micropores based on the transformation of coal’s molecular structure is proposed due to the chemical properties and difficult-to-transform properties of micropores. Carbon disulfide (CS_2_) extraction is used as a targeted stimulation to reveal the internal evolution mechanism of micropore transformation. The variations of microcrystalline structures and micropores of bituminous coal and anthracite extracted by CS_2_ were analyzed with X-ray diffraction (XRD), low-temperature carbon dioxide (CO_2_) adsorption, and molecular simulation. The results show that CS_2_ extraction, with the broken chain effect, swelling effect, and aromatic ring rearrangement effect, can promote micropore generation of bituminous coal by transforming the microcrystalline structure. Furthermore, CS_2_ extraction on bituminous coal can decrease the average micropore size and increase the micropore volume and area. The aromatic layer fragmentation effect of CS_2_ extraction on anthracite, compared to the micropore generation effect of the broken chain effect and swelling effect, can enlarge micropores more remarkably, as it induces an enhancement in the average micropore size and a decline in the micropore volume and area. The research is expected to provide a theoretical basis for establishing reservoir stimulation technology based on CS_2_ extraction.

## 1. Introduction

Coal is a classical porous medium containing fractures and pores of different sizes. These fractures and pores are the channels for coalbed methane (CBM) migration [1,2,3,4]. Pores primarily influence CBM desorption and diffusion capacity [5,6,7], while fractures mainly affect CBM seepage capacity [8,9,10]. Currently, most reservoir stimulation methods mainly focus on enhancing the permeability of coal by improving its fracture connectivity [11,12,13]. However, the adsorbed methane stored in micropores accounts for the total content of CBM (80–90%) [14,15,16]. Therefore, to further improve the CBM migration and development efficiency, more and more studies are paying attention to the pore stimulation of coal.

A series of reservoir stimulation technologies, mainly including hydraulic fracturing [17,18], CO_2_ phase change fracturing [19,20,21], electrical pulse [22], liquid nitrogen freeze–thaw [23], acid treatment [24,25], supercritical CO_2_ extraction [26,27], and solvent extraction [28], have been explored to transform pore connectivity. These technologies exert different effects on pore transformation due to their different action mechanisms. Furthermore, the pores with different sizes are divided into micropores, mesopores, and macropores based on the IUPAC [29]. Although hydraulic fracturing can transform mesopores and macropores [30], its water lock effect is inevitable. CO_2_ phase change fracturing only has an excellent transformation effect on mesopores and macropores rather than micropores [31,32]. Electrical pulse and liquid nitrogen freeze–thaw are only effective for pores with sizes of over 100 nm [33,34]. In short, these physical stimulation methods underperform in transforming micropores with chemical properties [35,36]. Also, acid treatment fails to exhibit any noticeable transformation effects on micropores by dissolving minerals in coal [37]. As for supercritical CO_2_ extraction, although it can transform micropores by extracting those small coal molecules over a long time (240 h), its influence on micropores with sizes of below 0.46 nm is relatively weak [38]. Besides, supercritical CO_2_ extraction requires rigorous temperature and pressure conditions to transform micropores [39].

By contrast, solvent extraction is superior in transforming micropores of coal because it can remarkably transform the coal molecular structure [40,41], and the walls of the micropores are made of atoms in the molecular structure of coal [36]. Therefore, micropores in coal have difficult-to-transform properties and chemical properties. Carbon disulfide (CS_2_), N-methylpyrrolidone, tetrahydrofuran, etc., are commonly applied to extract coal [42,43]. Therefore, the targeted stimulation of micropores based on the transformation of coal’s molecular structure is proposed because of the difficult-to-transform properties and chemical properties of micropores. The solvent extraction is used as a targeted stimulation to reveal the internal evolution mechanism of micropore transformation. The coal with different coalification indicates significant differences in terms of micropore and molecular structure [44,45], and different solvents also exert different extraction effects on them. Our previous research indicated that CS_2_ extraction on anthracite can increase the microfracture volume and permeability [46], but there is still a lack of comprehensive understanding of the evolution characteristics and mechanism on micropores of bituminous coal and anthracite by CS_2_ extraction targeted stimulation, which restricts the application of CS_2_ extraction in reservoir stimulation.

In this paper, CS_2_ extraction experiments were conducted on bituminous coal and anthracite. Next, the effects of CS_2_ extraction on the microcrystalline and micropore structures of the two coals were analyzed with the aid of XRD, low-temperature CO_2_ adsorption, and molecular structure simulation. Moreover, the stimulation mechanism of CS_2_ extraction on the micropore structure of coal was further revealed. The research is expected to provide a theoretical basis for establishing reservoir stimulation technology based on CS_2_ extraction.

## 2. Experiment and Methodology

### 2.1. Samples

The samples used in the experiments were bituminous coal from Pingdingshan No. 8 Coal Mine, named PDS, and anthracite from Zhongma Coal Mine in Jiaozuo, Henan Province, China, named JZ. After being collected, they were sealed for the subsequent testing and analysis. The basic analyses of the coal are listed in Table 1. The basic analyses indicate that PDS and JZ belong to bituminous coal and anthracite, respectively.

### 2.2. CS_2_ Extraction Experiment

**Figure 1:**
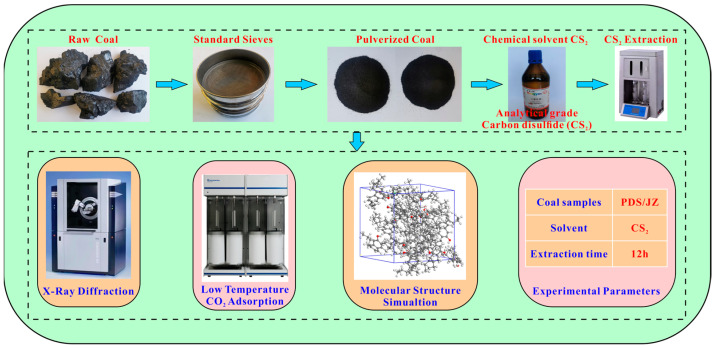
Flow chart of the experimental and measuring process.

The raw coal (PDS and JZ) was pulverized into 60–80 mesh and dried at 105 °C for 12 h. Then, it was subjected to the CS_2_ extraction experiments with a soxhlet extractor. In the experiment, 10 mL CS_2_ solvent were consumed per gram of coal sample, and the extraction time lasted 12 h. After the CS_2_ solvent-extracted treatment, the extracted coal samples were labeled PDST and JZT. The extracted coal samples were placed in a drying oven at a drying temperature of 105 °C. The coal sample was weighed every four hours until the weight change of the coal samples was less than 0.01 g and the samples were considered dry to constant weight. The extracted coal was measured by XRD and low-temperature CO_2_ adsorption. The experimental and measuring procedures are shown in Figure 1.

### 2.3. XRD Measurement

**Figure 2:**
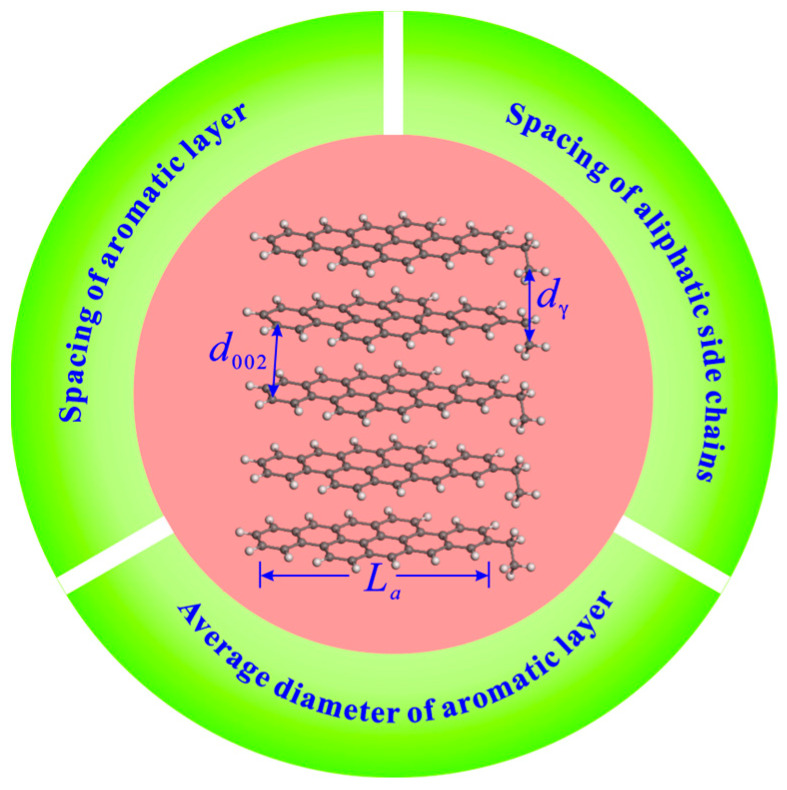
Diagram of microcrystalline structure parameters.

XRD measurement with copper Ka radiation (40 kV, 40 mA) was performed on raw and extracted coal samples with particle sizes of below 200 mesh by a D8 Advance X-ray diffractometer. The scanning range was 10–80°, the scanning speed was 2.0° per minute, and the step size was 0.02°. The coal microcrystalline parameters, including the chain spacing of aliphatic side chains (*d_γ_*), spacing of aromatic layers (*d*_002_), and average diameter of aromatic layers (*L_a_*), can be obtained through the XRD analysis (Figure 2). The calculation formulas of these parameters are follows.

The parameters *d_γ_* and *d*_002_ can be calculated from the positions of peak *γ* and peak 002 by Equations (1) and (2), respectively [46,47,48].

(1) $$d_{\gamma }=\frac{\lambda }{2\sin\theta_{\gamma }}$$

(2) $$d_{002}=\frac{\lambda }{2\sin\theta_{002}}$$

where *λ* is the X-ray wavelength, and *θ*_002_ and *θ_γ_* are the peak positions of peak 002 and peak *γ*.

The relevant research has demonstrated that the parameter *L_a_* can also be approximately calculated by Equation (3) [49,50].

(3) $$L_{a}=\frac{K_{a}\lambda }{B_{a}\cos\theta_{100}}$$

where *B_a_* is the full width at the half maximum of peak 100, and *K_a_* is 1.84 for peak 100.

### 2.4. Low-Temperature CO_2_ Adsorption Measurement

The low-temperature CO_2_ adsorption measurement was conducted on raw and extracted coal samples with sizes of 60–80 meshes at 273.15 K with the aid of an ASAP 2460 made by Micromeritics, Norcross, GA, USA. The micropore volume and area of the samples were estimated by the D-A model and the D-R model, respectively, and their pore diameter distribution was analyzed by DFT analysis [51].

### 2.5. Molecular Structure Simulation

Materials Studio 2019 software was employed to construct the conceptual models of coal’s molecular structure before and after CS_2_ extraction. The basic structural units of bituminous coal and anthracite, which were based on those of Wender et al. [52], were adjusted and modified by the Geometry Optimization and Anneal in Forcite module, which minimized the energy of the basic structural unit [53,54]. Furthermore, the conceptual models of bituminous coal and anthracite were constructed using the Amorphous Cell module and the modified basic structural units. Finally, micropores in the two coals were visualized using the Connolly surface [55,56,57].

## 3. Results

### Variation of Microcrystalline Structure Parameters

**Figure 3:**
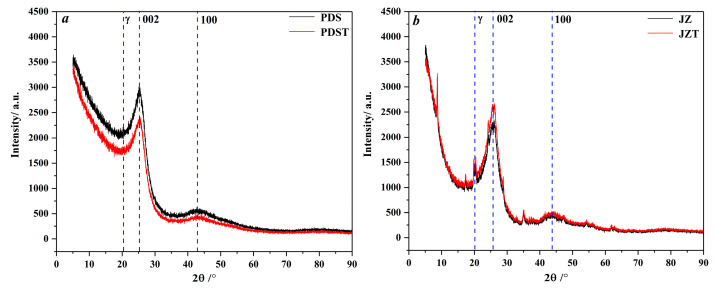
XRD spectra of raw and extracted coal: (**a**) PDS and PDST samples, (**b**) JZ and JZT samples.

As illustrated in Figure 3, peaks 002 and peak 100 of the raw and extracted coal are located in the angle (2*θ*) ranges of 20–30° and 40–50°, respectively. Peak *γ* is located to the left of peak 002 and overlaps with peak 002, which induces the asymmetrical shape of peak 002 [58,59,60].

**Figure 4:**
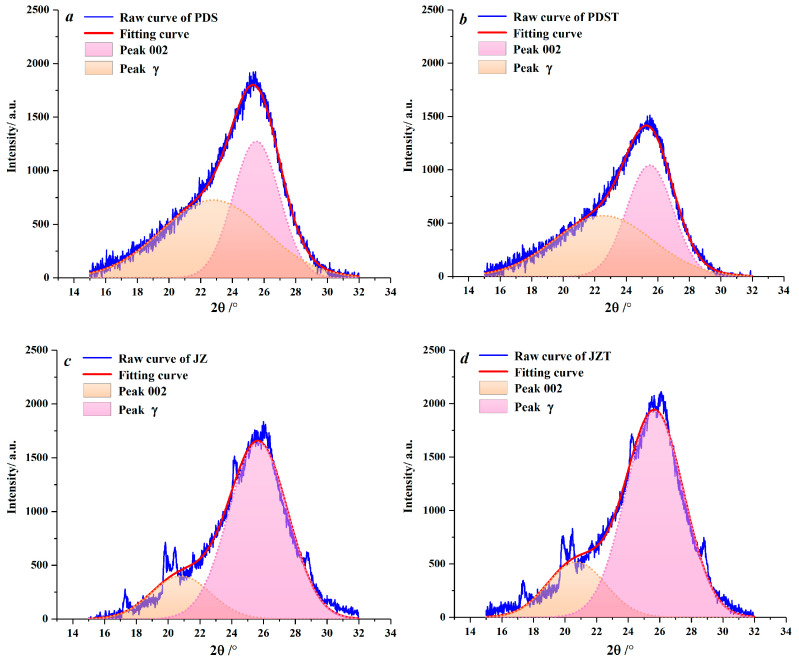
Peak fitting for peaks 002 and *γ*: (**a**) sample PDS, (**b**) sample PDST, (**c**) sample JZ, (**d**) sample JZT.

Peaks 002 and *γ* in XRD spectra were fitted by Peakfit 4.12 software (Figure 4). With he aid of this software, the microcrystalline parameters of the raw and extracted coal samples were derived (Table 2). Compared to PDS, JZ has larger *d_γ_* and *L_a_* values and smaller *d*_002_ values. During the coalification from bituminous coal to anthracite, the aliphatic side chains fall off [61,62], and the aromatic ring condensation effect becomes enhanced [63], which contributes to an increase in the *d_γ_* and *L_a_*. By combining with previous XRD and Raman spectroscopy studies of coal [64,65], the reduction in *d*_002_ reflects that the microcrystalline structure of coal continuously tends to graphitize during coalification [60,66].

**Figure 5:**
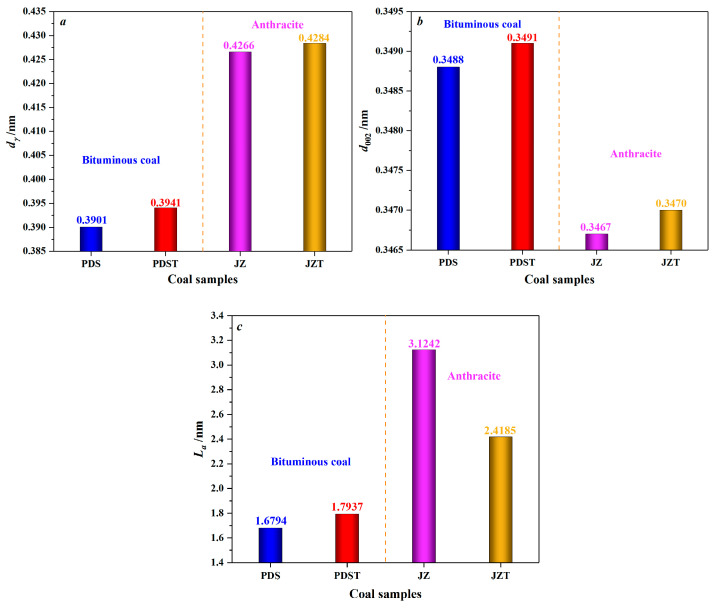
Variation in microcrystalline structure parameters of coal by CS_2_ extraction: (**a**) variation in the chain spacing of aliphatic side chains (*d_γ_*), (**b**) variation in the spacing of aromatic layers (*d*_002_), (**c**) variation in the average diameter of aromatic layers (*L_a_*).

As displayed in Figure 5, after being extracted by CS_2_, bituminous coal experiences an increase in *d_γ_* from 0.3901 to 0.3941 nm and an increase in *d*_002_ from 0.3488 to 0.3491 nm, indicating an enhancement in the spacing of aliphatic side chains and the spacing of aromatic layers. That is to say, CS_2_ extraction significantly reduces the microcrystalline structure cross-linking degree [67]. Moreover, after the CS_2_ extraction treatment, the *L_a_* value of bituminous coal increases from 1.6794 nm to 1.7937 nm. Such a result indicates that the extracted bituminous coal has larger aromatic layers and higher condensation of aromatic rings. The primary reason is that the low degree of cross-linking of the bituminous coal molecular structure around the second coalification jump [68] makes it easier for CS_2_ to contact the molecular structure fully. It is discovered that the CS_2_-extracted coal contains much more aliphatic hydrocarbons than aromatic hydrocarbons, from the previous analysis of its extracts [69]. This further reflects that CS_2_ will extract the aliphatic structures of coal preferentially. Moreover, bituminous coal has a large number of aliphatic structures [70,71]. Therefore, the extraction of aliphatic structures and partial aromatic structures can create favorable conditions for rearranging aromatic rings by reducing the degree of cross-linking of molecular structures [72] and increasing the *L_a_*.

As for anthracite, its *d_γ_* increases from 0.4266 to 0.4284 nm after CS_2_ extraction (Figure 5), which indicates an increase in the spacing of aliphatic side chains. However, such an increase is inferior to that of the *d_γ_* of CS_2_-extracted bituminous coal, mainly because anthracite has a low content of aliphatic structures [70,71]. Moreover, anthracite’s *d*_002_ increases from 0.3467 to 0.3470 nm after CS_2_ extraction, marking an increase in aromatic layer spacing. The decrease in *L_a_* of CS_2_-extracted anthracite reflects a reduction in the aromatic layers’ average diameter. In addition, the *L_a_* of THF-extracted anthracite also shows a decrease [67], but this decrease is less than that of CS_2_-extracted anthracite. What causes the above difference can be explained as follows: THF, a polar solvent, mainly affects the oxygen-containing functional groups [73,74], but anthracite, with a high degree of coalification, has few oxygen-containing functional groups and aliphatic structures [70,75]. By contrast, CS_2_, as a non-polar solvent [46], has excellent extraction and transformation effects on the aromatic structures of anthracite, causing a significant decrease in *L_a_*.

## 4. Discussion

### 4.1. Effects of CS_2_ Extraction on Microcrystalline Structure of Coal

**Figure 6:**
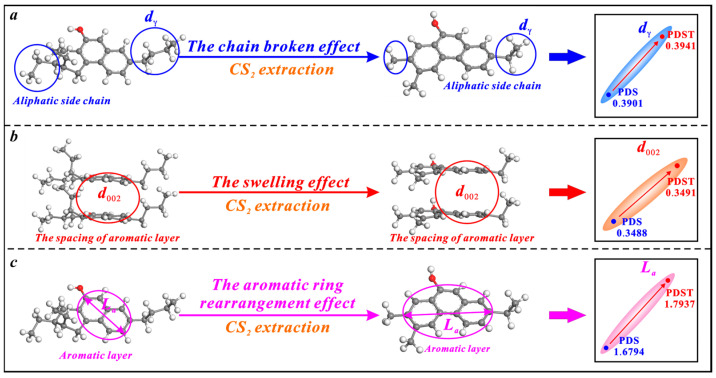
Effects of CS_2_ extraction on the microcrystalline structure of bituminous coal: (**a**) the broken chain effect, (**b**) the swelling effect, (**c**) the aromatic ring rearrangement effect.

CS_2_ extraction has triple effects on the microcrystalline structure of bituminous coal, i.e., the broken chain effect, swelling effect, and aromatic ring rearrangement effect (Figure 6). Bituminous coal contains a large number of chemical aliphatic structures [71,76], and CS_2_ extraction increases the *d_γ_* by breaking and shortening them (Figure 6a). The previous research demonstrates the irreversible swelling effect of cyclohexanone (CYC) extraction on the microcrystalline structure of bituminous coal. The molecules of CYC solvent tend to continuously penetrate the aromatic layers of coal and increase their spacing [77]. In this study, the solvent CS_2_ also displays an irreversible swelling effect on the microcrystalline structure of bituminous coal. When CS_2_ molecules penetrate the aromatic layers of bituminous coal, the *d*_002_ is promoted (Figure 6b). The microcrystalline structure cross-linking degree is reduced under the broken chain effect and the swelling effect, which causes an aromatic ring rearrangement effect to increase the *L_a_* (Figure 6c).

**Figure 7:**
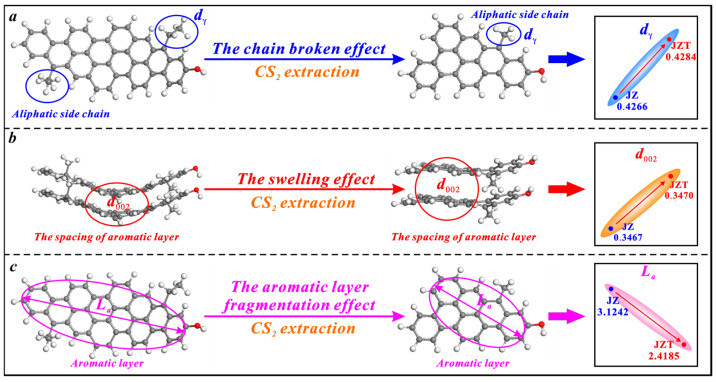
Effects of CS_2_ extraction on the microcrystalline structure of anthracite: (**a**) the broken chain effect, (**b**) the swelling effect, (**c**) the aromatic layer fragmentation effect.

CS_2_ extraction also has triple effects on the microcrystalline structure of anthracite, i.e., the broken chain effect, swelling effect, and aromatic layer fragmentation effect (Figure 7). However, its broken chain effect on anthracite is weaker than on bituminous coal. The effect only causes a slight increase in the spacing of anthracite’s aliphatic side chains (*d_γ_*) (Figure 7a), since anthracite contains fewer aliphatic side chains than bituminous coal [36,78]. Similarly, CS_2_ extraction causes an irreversible swelling effect on anthracite to increase the spacing of its aromatic layers (*d*_002_) (Figure 7b). Most of the CS_2_ molecules strongly extract the aromatic layers of anthracite due to its low content of aliphatic structures and further fragment the aromatic layers. Ultimately, this causes a decrease in the average diameter of anthracite’s aromatic layers (*L_a_*) (Figure 7c).

### 4.2. Effects of CS_2_ Extraction on Micropores

**Figure 8:**
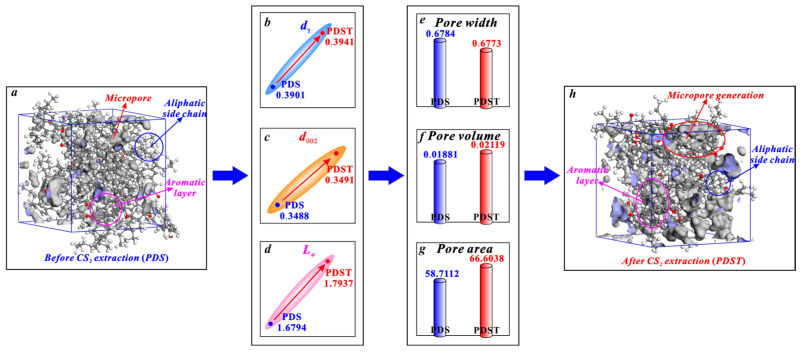
Effects of CS_2_ extraction on micropores of bituminous coal: (**a**) molecular structure of coal sample PDS, (**b**) variation in *d_γ_*, (**c**) variation in *d*_002_, (**d**) variation in *L_a_*, (**e**) variation in average pore width, (**f**) variation in pore volume, (**g**) variation in pore area, (**h**) molecular structure of coal sample PDST.

The micropores of bituminous coal mainly arise from the spacing between the aliphatic structure and aromatic structure (Figure 8). CS_2_ extraction enlarges the spacing of aliphatic side chains (*d_γ_*) with its broken chain effect (Figure 8b) and enlarges the spacing of aromatic layers (*d*_002_) with its swelling effect (Figure 8c), providing space for micropore generation [38,79]. Moreover, with its aromatic ring rearrangement effect, CS_2_ extraction increases the average diameter of aromatic layers (*L_a_*) (Figure 8d). This promotes the generation of micropores since micropores are mostly formed between the layers of two microcrystallites [36,80]. In this way, the above triple effects of CS_2_ extraction jointly promote the micropore generation of bituminous coal, among which the aromatic ring rearrangement effect plays a dominant role in controlling micropore generation. In detail, after a CS_2_ extraction treatment, bituminous coal experiences an average pore width decrease from 0.6784 to 0.6773 nm (Figure 8e), a micropore volume growth from 0.01881 to 0.02119 cm^3^/g (Figure 8f), and a micropore area growth from 58.7112 to 66.6038 m^2^/g (Figure 8g).

**Figure 9:**
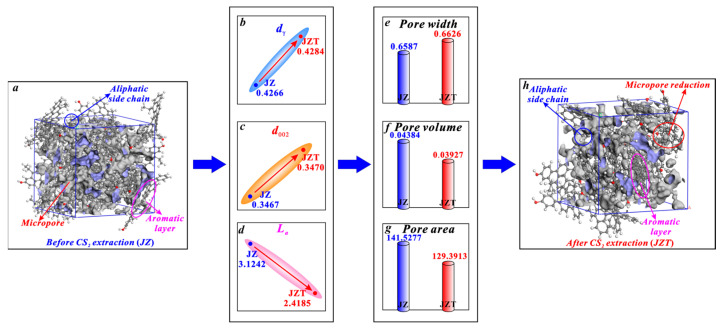
Effects of CS_2_ extraction on micropores of anthracite: (**a**) molecular structure of coal sample JZ, (**b**) variation in *d_γ_*, (**c**) variation in *d*_002_, (**d**) variation in *L_a_*, (**e**) variation in average pore width, (**f**) variation in pore volume, (**g**) variation in pore area, (**h**) molecular structure of coal sample JZT.

It can be seen in Figure 9 that the micropores of anthracite mainly arise from the spacing between aromatic layers. Similarly, according to the research of Liu et al. [36] on micropore evolution with coalification, the micropores of anthracite are mainly controlled by aromatic structures. First, it is noteworthy that anthracite contains few aliphatic structures [61,78], which are not the main structure for micropore generation in anthracite. Therefore, the increase in the *d_γ_* of anthracite (Figure 9b) caused by the broken chain effect cannot significantly promote micropore generation. Although the increase in the spacing of its aromatic layers (*d*_002_) (Figure 9c) caused by the swelling effect can promote micropore generation, the increasing rate of its *d*_002_ (0.0864%) is far less than the decreasing rate of its *L_a_* (22.5881%) after CS_2_ extraction. Noticeably, micropores are mostly formed between the layers of two microcrystallites [36,80]. Furthermore, Alvira et al. [81] concluded that fragmenting large carbon layers into small sheets produces a mesoporous structure. Similarly, the aromatic layer fragmentation effect will decrease the *L_a_* (Figure 9d) and thus improve the mesopore generation. Zhang et al. [46] put forward that the mesopores of anthracite become larger in volume after a CS_2_ extraction. Therefore, the reduction in *L_a_* caused by the aromatic layer fragmentation effect can promote the transformation of micropores into mesopores, which causes a reduction in the micropore number and an enlargement in the micropore size. Obviously, this micropore enlargement effect caused by the aromatic layer fragmentation effect is stronger than the micropore generation effect by the broken chain effect and the swelling effect. The aromatic layer fragmentation effect plays a dominant role in controlling the micropore enlargement. Therefore, it is the micropore enlargement effect that causes the variation in the structure parameters of anthracite micropores. In detail, after being extracted by CS_2_, anthracite undergoes an average pore width increase from 0.6587 to 0.6626 nm (Figure 9e), a micropore volume decrease from 0.04384 to 0.03927 cm^3^/g (Figure 9f), and a micropore area decrease from 141.5277 to 125.3913 m^2^/g (Figure 9g).

### 4.3. Implication and Limitation

Our recent research analyzed the micropore (<2 nm), mesopore (2–50 nm), and macropore (>50 nm) variation characteristics of CS_2_-extracted anthracite. The macropore and mesopore volume increased significantly, while the micropore volume decreased [82]. However, the mechanism by which the microcrystalline structure evolution of CS_2_-extracted coal controls the evolution of micropores has not been effectively revealed, especially for bituminous coal. This study explored the triple effects of CS_2_ extraction on the microcrystalline structures of bituminous coal (i.e., the broken chain effect, swelling effect, and aromatic ring rearrangement effect) and anthracite (i.e., the broken chain effect, swelling effect, and aromatic layer fragmentation effect). Moreover, it also investigated the micropore generation effect of CS_2_ extraction on bituminous coal and the micropore enlargement effect of it on anthracite. The results revealed that CS_2_ extraction boasts unique advantages of chemical transformation of micropores compared with conventional reservoir stimulation technologies, including hydraulic fracturing, CO_2_ phase change fracturing (CO_2_-PTF), liquid nitrogen freeze–thaw, etc.

**Figure 10:**
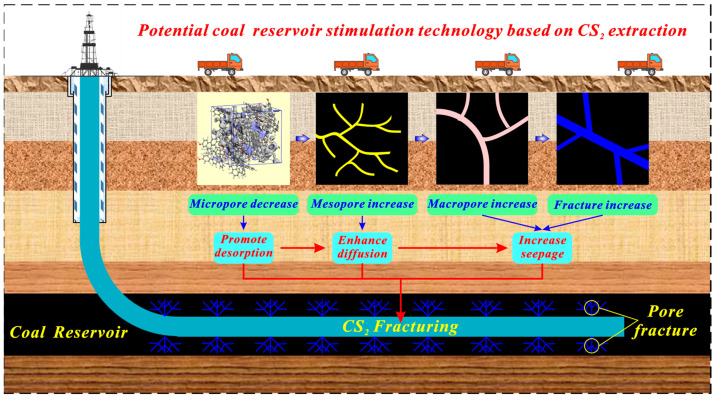
Diagram of potential reservoir stimulation technology based on CS_2_ extraction.

Figure 10 indicates the diagram of potential reservoir stimulation technology–CS_2_ fracturing of a horizontal well in anthracite reservoirs. Coal reservoirs contain multi-scale pore and fracture structures, which influence the desorption–diffusion–seepage capabilities of coalbed methane [83]. The micropore is the major space where CBM is adsorbed and stored [84,85]. CS_2_ extraction, with a micropore enlargement effect on anthracite, can increase its average micropore size and decrease its micropore volume and area, which is conducive to gas desorption. Besides, as proven in our previous research, CS_2_ extraction can increase the mesopore and macropore volume significantly and generate a large number of microfractures in anthracite [46,82], which is conducive to gas diffusion and seepage. Therefore, CS_2_ extraction is conducive to “promote desorption–enhance diffusion–increase seepage” for improving the gas migration ability for anthracite. Further, CS_2_ can be considered the fracturing fluid to establish the novel reservoir stimulation technology, which has effective transformation and stimulation effects to avoid the water-locking effect of water-based fracturing fluid. However, the micropore generation effect of CS_2_ extraction on bituminous coal may enhance the adsorption capacity of coal reservoirs, which may be not conducive to gas desorption. Significantly, our previous study demonstrated that CS_2_ transformation microfractures in coal reservoirs have a time effect [46]. Extending the extraction time for CS_2_ to transform micropores in bituminous coal may have a potential micropore enlargement effect. Such a transformation effect on bituminous coal remains uncertain and requires further research.

**Figure 11:**
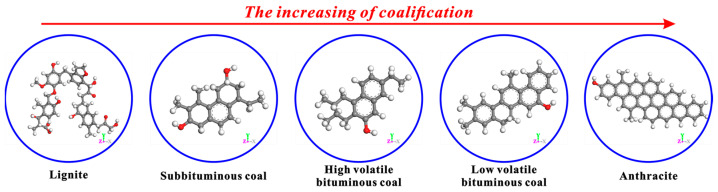
Basic structural units of coals with different coalification degrees [52].

In addition, the apparent differences in the basic structural units of coals with different coalification degrees can be observed in Figure 11. From lignite to anthracite, the basic structural unit of coal’s molecular structure shows regular changes with the increase in coalification degree. Specifically, the numbers of side chains and functional groups keep decreasing, and that of condensed aromatic rings keeps increasing. In the anthracite stage, the condensed aromatic rings mount rapidly, while the side chains and functional groups almost disappear. In this study, only the effects of CS_2_ extraction on the microcrystalline structures and micropores of bituminous coal and anthracite were discussed. In fact, the influence of CS_2_ extraction on the microcrystalline structures and micropores of other coals with different coalification degrees needs to be further explored.

## 5. Conclusions

This research discussed the influence of CS_2_ extraction targeted stimulation on the microcrystalline and micropore structures of bituminous coal and anthracite. The related conclusions are as follows:

(1) CS_2_ extraction exerts a broken chain effect, swelling effect, and aromatic ring rearrangement effect on the microcrystalline structure of bituminous coal, increasing the spacing of its aliphatic side chains (*d_γ_*), the spacing of its aromatic layers (*d*_002_), and the average diameter of its aromatic layers (*L_a_*).

(2) CS_2_ extraction exerts a broken chain effect, swelling effect, and aromatic layer fragmentation effect on the microcrystalline structure of anthracite, increasing the spacing of its aliphatic side chains (*d_γ_*) and the spacing of its aromatic layers (*d*_002_), and reducing the average diameter of its aromatic layers (*L_a_*).

(3) CS_2_ extraction, which has a micropore generation effect on bituminous coal, can decrease its average micropore size and significantly increase its micropore volume and area.

(4) The micropore enlargement effect caused by the aromatic layer fragmentation effect on CS_2_-extracted anthracite, which is prominently stronger than the micropore generation effect caused by the broken chain effect and the swelling effect, can expand the average micropore size and significantly reduce the micropore volume and area.

(5) This research provides a theoretical reference for improving reservoir stimulation technology on micropores in coal reservoirs based on CS_2_ extraction.

## Figures and Tables

**Table 1:** Results of the basic analyses on coal samples.

Coal	R_o,max_, %	Proximate Analysis, %				Elemental Analysis, %				
		M_ad_	A_ad_	V_daf_	FC_ad_	C	H	O	N	S
PDS	1.22	0.97	10.03	24.91	66.83	77.05	4.34	5.40	1.15	0.37
JZ	2.94	2.94	8.41	5.50	83.15	93.27	3.05	2.29	1.09	0.30

Note: R_o,max_ is the vitrinite maximum reflectance, M_ad_ is the air dry moisture content, A_ad_ is the air dry ash yield, V_daf_ is the dry ash free volatile matter, and FC_ad_ is the air dry fixed carbon.

**Table 2:** Microcrystalline parameters of raw and extracted coal.

Samples	*θ_γ_*/°	*θ*_002_/°	*θ*_100_/°	*d_γ_*/nm	*d*_002_/nm	*L_a_*/nm
PDS	11.3866	12.7567	22.0506	0.3901	0.3488	1.6794
PDST	11.2696	12.7454	22.1817	0.3941	0.3491	1.7937
JZ	10.4022	12.8373	22.0453	0.4266	0.3467	3.1242
JZT	10.3586	12.8253	21.8877	0.4284	0.3470	2.4185

## Data Availability

Data are contained within the article.
